# Multiscale Fine-Grained Heart Rate Variability Analysis for Recognizing the Severity of Hypertension

**DOI:** 10.1155/2019/4936179

**Published:** 2019-01-22

**Authors:** Hongbo Ni, Ying Wang, Guoxing Xu, Ziqiang Shao, Wei Zhang, Xingshe Zhou

**Affiliations:** School of Computer Science, Northwestern Polytechnical University, Xi'an 710072, China

## Abstract

Hypertension is a common and chronic disease and causes severe damage to patients' health. Blood pressure of a human being is controlled by the autonomic nervous system. Heart rate variability (HRV) is an impact of the autonomic nervous system and an indicator of the balance of the cardiac sympathetic nerve and vagus nerve. HRV is a good method to recognize the severity of hypertension due to the specificity for prediction. In this paper, we proposed a novel fine-grained HRV analysis method to enhance the precision of recognition. In order to analyze the HRV of the patient, we segment the overnight electrocardiogram (ECG) into various scales. 18 HRV multidimensional features in the time, frequency, and nonlinear domain are extracted, and then the temporal pyramid pooling method is designed to reduce feature dimensions. Multifactor analysis of variance (MANOVA) is applied to filter the related features and establish the hypertension recognizing model with relevant features to efficiently recognize the patients' severity. In this paper, 139 hypertension patients' real clinical ECG data are applied, and the overall precision is 95.1%. The experimental results validate the effectiveness and reliability of the proposed recognition method in the work.

## 1. Introduction

Hypertension is a common and chronic disease, and the hypertensive patients have no symptom when it is in the early stage. At present, the most prominent problems of the treatment of hypertension are low awareness, low treatment, and low control. It is highly regarded as the dangerous healthy problem throughout the world because of its high prevalence and its association with increased risk of cardiovascular disease. Nowadays, hypertension is the leading cause of death in the world [1]. In China, 70% patients do not know they have suffered from hypertension, and even 75% patients were not treated, and also 90% patients' condition was not controlled. Therefore, it is a challenging problem how to improve the awareness, treatment, and control rates of hypertension. HRV reflects the autonomic nervous system activity and quantitative evaluation of cardiac sympathetic nerve and vagus nerve tension and balance, to determine the washing is an important indicator of cardiovascular disease. ECG is an effective measure and records the details of the electrical activity of diagnostic equipment [2], and ECG signals including the ventricular electrical activation of *Q*, *R*, and *S* waves. Heart rate varies with each cardiac signal, HRV is a time period between heart rate, which is also called the RR intervals. Moreover, there are a number of wearable and nonintrusive devices to monitor ECG and HRV; for example, Sinabro monitors ECG during smartphone use, by leveraging sensors integrated into the phone [3]. Electrodes have also been directly embedded into a bed to continuously sense presence, position, and ECG [4].

Meanwhile, one-third of one's life time is under a state of sleep. Sleep is an important physical phenomenon, which is an essential physiological process in the human's daily life. Many kinds of cardiovascular diseases may have a different symptom compared with daytime, including stroke and myocardial infarction. And in the sleep state, the body's signs are stable and respond more intuitive for the severity of the disease.

Blood pressure measurement has always been the golden standard to diagnose hypertension in clinic environment. There are diverse simple domestic sphygmomanometers, for common people to monitor their blood pressure, and the users should follow the particular instructions intentionally with correct gesture, at right time and keeping calm. Fortunately, monitoring HRV is a new alternative approach for hypertension, and the users can monitor their healthy status in a nonintrusive manner, for example, during their natural sleep.

Based on the above statements, it is a reasonable way to recognize the severity of hypertension by HRV extracted from the ECG in overnight sleep. HRV analysis is usually classified into long-term analysis and short-term analysis. In general, long-term analysis is often a 24-hour analysis, including all activities of human being all day, such as eating, working, and sleeping; short-term analysis is an analyzing of 5 minutes HRV data. To bridge the gap between long-term and short-term analysis, a fine-grained analysis method is needed in practical work.

In summary, the contributions in this paper are in three aspects:

Firstly, we present a novel data processing method based on various time window dividing modes on the overnight data. Secondly, we propose a feature dimension reduction method called the temporal pyramid pooling method. Finally, we employ multifactor analysis of the variance-based feature selection method and establish a hypertension degree recognizing model. In a word, we propose a fine-grained HRV analysis method to enhance the precision of the severity of hypertension recognition.

The rest of the paper is organized as follows: we discuss related works in Section 2. In Section 3, analysis flow in this research is presented. We show the data processing method in Section 4.  Section 5 addresses the details of the fine-grained HRV analysis method. Finally, Section 6 discusses the experiments, and then we conclude the paper in Section 7.

## 2. Related Work

HRV is the variability of every heartbeat intervals and reflects the autonomic nervous system activity and quantitative evaluation of the cardiac sympathetic nerve and the tension of the vagus nerve and balance of the cardiac sympathetic and vagus nerves. And HRV analysis is also a reliable reflection of the many physiological factors modulating the normal rhythm of the heart [2]. Measurements of HRV are nonintrusive and easy to perform, have relatively good reproducibility, and provide prognostic information for daily health care and chronic diseases.

Many methods are applied to monitor HRV, such as photoplethysmography (PPG) [3], ECG [5, 6], and ballistocardiogram (BCG) [7–9]. ECG measures the electrical activity of the heart while the heartbeats. ECG is a diagnostic tool that measures and records the electrical activity of the heart in detail, and HRV can be accurately extracted from ECG [2].

HRV is affected by many cardiovascular risk factors such as hypertension, and investigations have revealed that depressed HRV is associated with cardiac, vascular, and renal target organ damage in hypertensive patients. So HRV is a simple noninvasive measurement method to research the influence of the cardiovascular system [10]. A lot of researches focus on the relationship of HRV and hypertension. Thomas found difference in the complexity of HRV in normal pregnant people and hypertensive pregnant people [11]. The HRV in patients with masked hypertension (MH) was observed using a 24-hour Holter monitor, and the changes of time-domain indicators showed cardiac autonomic nerve dysfunction in patients with masked hypertension [5]. Moreover, some researchers discuss the relationship between normal people and hypertension patient [6, 12, 13]. Natarajan shows that the condition of hypertension will go worse while the parameter of HRV in time domain and frequency domain decreases [12]. Investigations discussed the relationship between normal and untreated hypertensive patients, and also the results showed all time and frequency domain heart rate variability parameters were reduced and also deteriorated in the hypertensive population [13]. Ni recognized normal people and hypertension patients with the predictable and sensitive specificity of HRV [6].

Meanwhile, many researchers combine HRV and machine learning method to diagnose hypertension [6, 13, 14]. A novel predictive model based on machine learning algorithms was developed to provide an automatic risk stratification tool for hypertensive patients [12]. Poddar combines linear HRV features and nonlinear features to recognize normal people and hypertension patients [13]. Another research can automatically recognize hypertensive patients from normal people with continuous HRV monitoring overnight with the three-dimensional features [6]. Mussalo focus on the detection of the severity of hypertension, and there are distinct differences in HRV features while the hypertension patients are in the mild stage or severe stage [14]. Medically, the severity of hypertension is often classified into mild, moderate, and severe. It is realistic to recognize the severity of hypertension in more fine-grained analysis.

## 3. Analysis Flow of the Research

**Figure 1:**
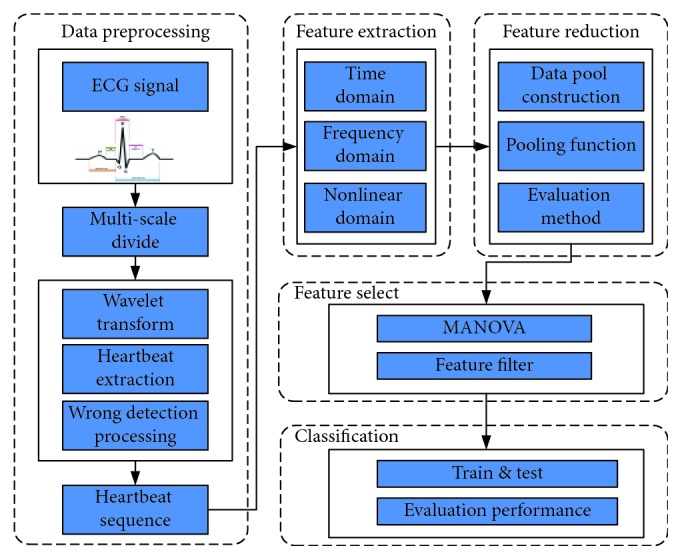
Analysis flow of the research.

In this work, the analysis flow can be seen in Figure 1. Firstly, the raw ECG signals are divided into diverse scales. Wavelet transform is to filter the signal, and peak detection is to get the primary heartbeat sequence. Secondly, an error detecting method is used to obtain the final correct heartbeat sequence. According to the HRV features extracted from diverse scales, the authors design a pooling approach to reduce the dimension of the feature vector. Finally, with the MANOVA method filtering the relevant features, a severity model for recognizing hypertension based on refined features is proposed.

## 4. Data Preprocessing

### 4.1. Multiscale Division

As mentioned in the previous section, there are two methods of HRV analysis, that is, the long-term analysis and short-term analysis. Both of the methods regard the ECG as an indivisible whole and cannot reflect the subtle dynamics of HRV during the period. In order for fine-grained analysis, we cut the data into different time scales.

In this work, we applied the dataset of SHAREE (Smart Health for Assessing the Risk of Events via ECG) from Physionet, which includes 24-hour ECG data of 139 subjects [15], and at first, we extract the patients' ECG data in the sleeping stage from 10 pm to 6 am, i.e., 8 hours in total. When the patient is in the sleep state, the patients' HRV have no disturbance from external environment, and HRV can actually reflect the physiological status of the body, for hypertension patients, and accurately reflect the severity of the illness. Furthermore, we cut the 8 hours long-term data into 4 hours, 2 hours, 1 hour, and half an hour. In consideration of 5 minutes and 20 minutes time scale often applied in some study of recognizing diseases, we also add these two scales in the work.

### 4.2. Wavelet Transform

Wavelet transform has the characteristics of multiresolution analysis, which is also known as a multiscale feature [16]. Wavelet transform is similar to a binary filter which has high pass and low pass filters at the same time. As a result, wavelet transform can also be used for filtering. After wavelet transform, we, respectively, obtain low-frequency contour information and high-frequency mutations, and these features describe the distribution characteristics on both time domain and frequency domain. Based on the specificity, we are able to analyze the time-frequency domain features, which mean that the characteristics observed on a certain resolution can be easily observed on another resolution. So, the signal characteristics under a resolution can be observed based on wavelet decomposition.

### 4.3. Heart Rate Intervals Extraction

Heart rate intervals is the basis of research cardiovascular diseases using HRV. In this paper, intervals between R peaks refer to the RR interval. So, it is essential for us to detect R peaks from ECG or BCG. Then, it is simple to calculate the difference between the adjacent heartbeats, and this is also called heart rate time series. In order to extract accurate heart rate intervals, researchers have proposed various methods, including threshold method, template matching method, and sliding window. In this paper, we use fixed sliding window to extract the heart rate intervals to recognize all the R peaks in ECG [7].

### 4.4. Classification Tags

In 2003, the European society of hypertension (ESH) and the European society of cardiology (ESC) established hypertension guidelines and get the recognition of the international union of high blood pressure. In June 2007, ESH released the new hypertension guidelines because a lot of clinical new evidence appeared from the experiment and treatment of hypertension after the guidelines issued in 2003. The new guidelines emphasize the flexibility of real hypertension threshold, which should be decided by cardiovascular risk factors. The ECG data are recorded by the patients recruited at the Centre of Hypertension of the University Hospital of Naples Federico II, Naples, Italy. Therefore, in this work, we use the ESH guidelines to mark tags, as seen in Table 1. Due to the flexibility of risks of hypertension, we use the ESH guidelines to redivide the risks of hypertension. We mark tags to the patients by the hospital according to the ESH guidelines. The ECG Holter was performed after a one-month antihypertensive therapy washout. Therefore, the patients are divided into mild, normal hypertension, and primary hypertension patients, and also in primary patients, level I is divided into moderate, level 2 and level 3 hypertension patients are divided into severe.

## 5. Feature Extraction

### 5.1. HRV Features Extraction

#### 5.1.1. Time Domain

Time-domain analysis refers to the variability of adjacent normal RR intervals over a period of time. Mathematical and statistical analysis was performed by comparing the values of the RR interval in the order of time or heartbeat. We use the following time-domain features in this work:

- MEAN: the average of RR intervals during a period of time
- VAR: the variance of RR intervals during a period of time
- MAX: the longest RR interval during a period of time
- MIN: the shortest RR interval during a period of time
- SDNN: the standard deviation of RR intervals during a period of time
- RMSSD: the root mean square of difference between RR intervals during a period of time
- SDSD: the standard deviation of difference between RR intervals during a period of time
- PNN50: the percentage of the number of adjacent RR intervals which is more than 50 ms during a period of time
- CV: the degree of variation of HRV during a period of time, represented by ratio of SDNN and MEAN

Time-domain features can perfectly reflect the average-level and dispersion degree of RR intervals. But time-domain index cannot assess the autonomic nervous system and the balance of the sympathetic nerve and vagus nerve.

#### 5.1.2. Frequency Domain

Frequency-domain analysis is usually employed for short-term analysis, which usually takes 5 min ECG recording to analysis. Using fast Fourier transform or autoregressive analysis, technology obtains spectrum from the ECG signal, and frequency-domain features are divided into high-frequency power (0.15∼0.40 Hz) and low-frequency power (0.04∼0.15 Hz). High frequency power responses vagal adjustment function, respiratory sinus arrhythmia, high frequency power peak with changes in respiratory rate changes, and the peak amplitude affected by respiration. The low frequency power is related to the pressure reflex regulation, which reflects the complex regulation of the sympathetic and parasympathetic nervous system on the sinus node. In addition, spectral standardization and low-frequency power/high-frequency power processing methods have also begun to evaluate the sympathetic and vagal balance.

In the frequency domain, we use the following features:

- VLF: very low-frequency power (0.003∼0.04 Hz)
- LF: low-frequency power (0.04∼0.15 Hz)
- HF: high-frequency power (0.15∼0.40 Hz)
- VHF: very high-frequency power (0.40∼0.50 HZ)
- TP: total power
- Low-frequency power/high-frequency power (LF/HF)

#### 5.1.3. Nonlinear Domain

The biological system is a complex chaotic system because the heartbeats are dominated by the autonomic nervous system, and brain activity affects autonomic nervous activity, and also brain activity indirectly affects or dominates cardiac activity. The brain's activity can be seen as a complex dynamic system of multiple nonlinear oscillators due to interaction. Therefore, the complexity analysis parameters can be used to reflect the dynamic characteristics of heart rate variability (HRV), which in turn reflects on the heart condition. Recently, nonlinear dynamics method has a large number of applications in quantitative analysis of HRV signals. In the nonlinear domain, we use DFA, Renyi entropy, and sample entropy as nonlinear features.

### 5.2. Feature Dimension Reduction

Feature dimension reduction refers to the feature in the collection from the initial high dimension and the optimization to reduce the feature space, resulting in a lower dimensional feature space. Feature dimension reduction is usually a machine learning preprocessing step, which can effectively eliminate the irrelevant and redundant features, and improve the efficiency and the performance of the model of machine learning. In this work, in order to perform fine-grained HRV analysis, we cut the ECG data into different scales. And this causes the problem of data dimension uprush. In this work, we design a temporal pyramid pooling [16] method to reduce the dimension of features. Temporal pyramid pooling contains data pool construction, pooling function, and evaluation methods.

#### 5.2.1. Data Pool Construction

Temporal feature dimension reduction method needs to stack the sequence features to increase the dimension of the input before the feature processing. In this work, we need to stack the feature to form the data pool. We separate the data into different scales which cause the feature dimension explosion. And we also have two methods to form the data pool, single scale, or multiscales.

*Single Scale*. In this work, we have a comprehensive analysis to the patients' ECG data in the time domain, frequency domain, and nonlinear domain and then extract 18 features. We stack every feature in the same time scales to form a single-scale data pool. So we get 18 feature pools in every time scale and then use pooling function to reduce the dimension of the feature vector.

**Figure 2:**
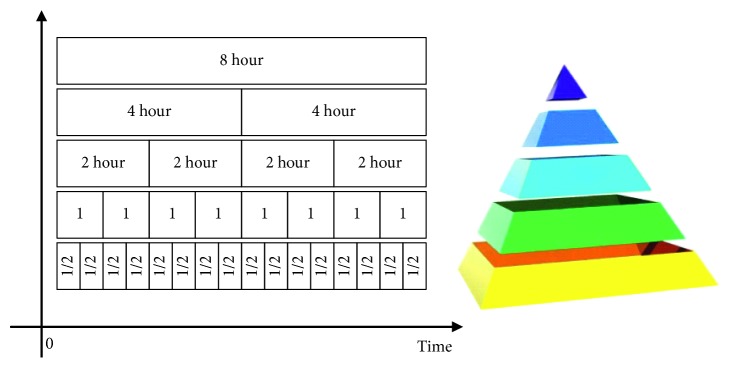
Temporal pyramid data pooling.

*Temporal Pyramid Data Pooling*. Pooling method is first used in the image processing field. Pictures can be represented as an *n* ∗ m matrix. So in image processing field, data pool is in existence. But in our work, we must firstly establish the data pool in an appropriate way. A temporal pyramid data pool was constructed in this work. The pyramid has 7 levels and stacks features of different scales, as seen in Table 2, the longest data segment is 8 hours, and the shortest data segment is 5 minutes. All of signals were segmented into different scales by the level 0 (8 hours) and 18 types of features which contains time-domain features, frequency-domain features, and nonlinear-domain features are extracted for each scale of signal. In this work, we construct temporal pyramid data pool for different features. Therefore, level 0 produces the single TD, FD, and EN features because the feature is extracted from the entire signal. Level 1 with 4-hour temporal resolution produces 2 features, and level 3 with 2 hour have 4 features. The number of feature dimensions are shown in Table 3. Then, we stack different number of features into a temporal pyramid data pool, as seen in Figure 2. As a result, we extract 151 features for every type of feature.

#### 5.2.2. Pooling Function

Pooling function is used to combine the features over different time scales using a statistical method in order to create the combined feature representation based on the temporal pyramid data pool. It makes invariance to small transformations or robustness to noise as well as compact representation. The typical pooling operation is sum, average, or maximum; sum pooling is to sum features over the region, average pooling takes the average of features from the subregions, and maximum pooling picks up the maximal feature response per subregion. But these methods will cause a lot of information loss. So we design a sequence-based pooling method to fuse features in different scales.

Every type of features construct a temporal pyramid pool, and the feature vector isT={*t*_1_, *t*_2_, *t*_3_,…, *t*_*n*_}, where *n* means the number of features. In this paper, as discussed in Table 3, *n* was 151. The pooling function is in the following steps:

Step 1. Sort the features, and the descending feature vector is *T*={*a*_1_, *a*_2_, *a*_3_,…, *a*_*n*_}.
Step 2. Select the top *k* features, and the weight of every feature is 1/*k*:

(1) $$\begin{array}{c} t=\frac{1}{k}\overset{k}{\underset{i=1}{\sum }}a_{i}. \end{array}$$

In this work, *k* means the threshold of features participated in the pooling.

The choice of *k* is an important problem, the value of *k* cannot be too large or too small. When *k* = 1, it is just a maximum pooling method, and when *k* = *n*, it is just an average pooling method. So in this work, we use evaluation methods to estimate the discriminative of fusing features when *k* is a different value.

#### 5.2.3. Evaluation Methods

In this work, information entropy is used to quantify the degree of uncertainty relative to the classification and determine the information of classification the feature contains.

For every feature, the feature vector is *X*_*k*_ when *k* is a different value. The information entropy of the feature vector is *H*(*X*_*k*_):

(2) $$\begin{array}{c} H\left(X_{k}\right)=-\int_{x}p\left(x\right)\log\;p\left(x\right)\;dx, \end{array}$$

where *p*(*x*) means the probability of *X*_*k*_ value for *x*. When the information entropy of the feature is higher, it shows its scope of even distribution and has higher existence of information; when the information entropy is lower, it shows the distribution is uneven, and a lot of samples take only one or a few values; and when the information entropy is zero, all of the sample is the same, and it cannot provide any useful information. In this work, we calculate the information entropy when *k* takes different values from 0 to 151 for each feature, and we select a right *k* value when the information entropy of the specific feature reaches the maximum, and different features have a corresponding *k* value, respectively. The results are in Table 4.

### 5.3. Feature Selection

**Figure 3:**
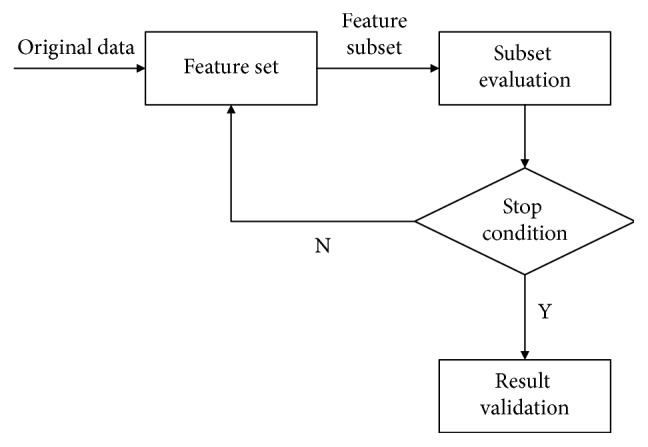
Basic framework of feature selection.

In general, feature selection is executed before the training of the classifier. Feature selection can effectively reduce the feature dimension and the difficulty of training tasks. Meanwhile, the classification model built on a small amount of relevant features has stronger interpretability [17]. Feature selection has two key steps, including subset searching and subset evaluation. The basic framework of feature selection is shown in Figure 3.

In this work, the severity of the patients with hypertension can be divided into mild, moderate, and severe. Using the basic statistical methods, such as *T*-test, we can only analyze the significance of any two classes and then choose the higher relevant features. So basic statistical methods cannot achieve the goal of multivariate analysis. Variance analysis can measure the difference between two or more classes at the same time. Therefore, it is important to evaluate the relationships between the various HRV features and their influence on the patients in different severity of hypertension. So, MANOVA is used to select relevant features and F-measure is used to measure the importance of the feature vector in this work.

While in feature extraction, we extract 18 features in the time domain, frequency domain, and nonlinear domain on the basis of previous work [6], including 9 features, 6 features, and 3 features, respectively. Thereby, MANOVA is used to filter the relevant features and remove the interference of irrelevant information on hypertension severity recognition.

Initially, we analyze the features of single-scale data pooling and compute the F-measure of features with MANOVA, and the result is in Table 5. In this work, we consider the F-measure which is greater than 1 is significant features. There are some common features: in the time domain, MAX, MEAN, and SDSD are included, and in the frequency domain, LF/HF, and VHF are included.

Then, MANOVA is used for temporal pyramid data pool, and the result is in Table 6, which shows the different F-measure of features, and we filter 9 features in this feature set whose F-measure is greater than 1. The feature vector is expanded based on the single data pool, and more features are selected in this experiment. In the time domain, MIN and RMSSD are added. In the frequency domain, HF, LF, and VLF are added. Feature selection method can effectively reduce the dimension of the feature vector and retain the beneficial information as much as possible. Therefore, the model of recognition severity of hypertension is built in a lower dimension of the feature vector and also with stronger interpretability and generalization ability.

## 6. Experiment

In this section, we adopt four classification methods to recognize the severity of hypertension, namely, Naïve Bayes, support vector machine (SVM), backpropagation neural network (BPNN), and random forest (RF) classifiers. The temporal pyramid pooling method is employed to reduce the dimension of the features vector. And the features selected by MANOVA is applied as an input for the classifier. We present the experimental results for various classifiers and a different processing of the feature vector.

### 6.1. Experiment Result

In this work, 10-fold cross validation is adopted to train the recognition models, and the performance indicators are defined as following:

(3) $$\begin{array}{c} \left\{\begin{array}{c} \text{precision}=\frac{\text{TP}}{\text{TP}+\text{FP}}\times 100,\\ \text{recall}=\frac{\text{TP}}{\text{TP}+\text{FN}}\times 100. \end{array}\right. \end{array}$$

### 6.2. Classifiers in the Experiments

**Figure 4:**
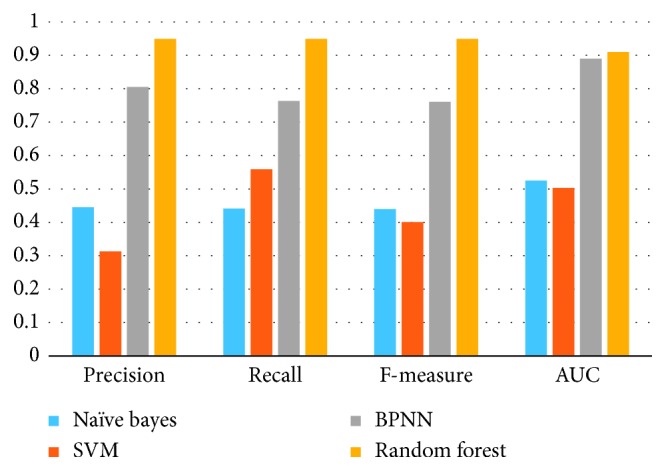
The result of classifiers.

At first, we use the unselected feature vector to train the recognition model to compare the performance of different classifiers. The result of this experiment is in Figure 4. In our experiment, random forest algorithm gets a better performance and reaches a precision of 94.9%. As known, most of classifiers are impacted by the balance of the dataset. When the dataset is unbalance, the performance of the classifier will be affected and the precision of recognition will be greatly reduced.

As seen in Table 7, Naïve Bayes method, BPNN method, and SVM method cannot adapt the unbalanced dataset. Random forest classifier is an easy convergence to the locally optimal solution and also offers higher tolerance for the noise and outlier from the dataset, which is suitable for processing the unbalanced and high dimension dataset. So in this work, RF methods are employed to construct models in subsequent experiments.

### 6.3. Experiment of Diverse Time Scales

**Figure 5:**
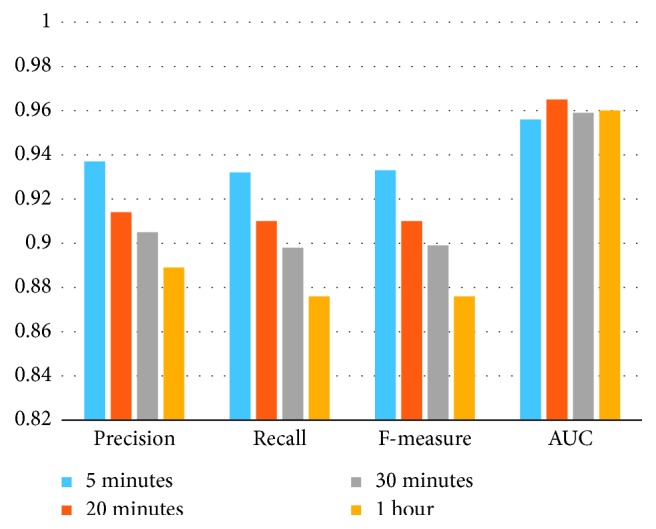
The result of different time scales.

Features extracted in single temporal scales are used as an input for the random forest classifier. The result of this experiment is in Figure 5. From the table, we can conclude that the performance of the RF classifier gets worse as the time scales increase. It means that fine-grained HRV analysis can improve the performance of the classifier. Comparing the single temporal scale with the temporal pyramid pooling method, the multiscale stack method has a better performance. Diverse feature has a different performance in various time scales, and the temporal pyramid pooling method has a more fine-grained analysis and therefore improves the performance of the classifier.

### 6.4. Experiment of MANOVA

**Figure 6:**
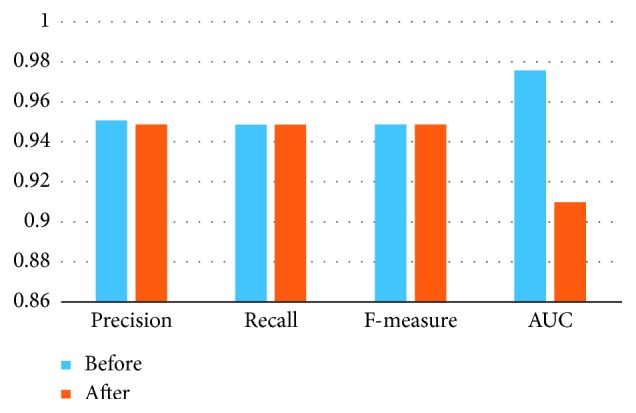
The result of feature selection.

At first, we extract 18 HRV features in different scales. Then, we use the temporal stack method to process these features. But not all of these features are useful for the classification. In this work, MANOVA method is applied to select relevant features and finally gets 9 relevant features. The result of this experiment is in Figure 6.

According to the performance of classification before and after feature selection, MANOVA method is an effective method to filter the features for recognizing hypertension. The performance of the classifier does not get worse with less features. The relevant features can more effectively reflect the physiological status of the patients, and the interpretability and the generalization capacity of the model built based on a lower dimension feature space increase greatly.

### 6.5. Experiment of Dimension Reduction Method

**Figure 7:**
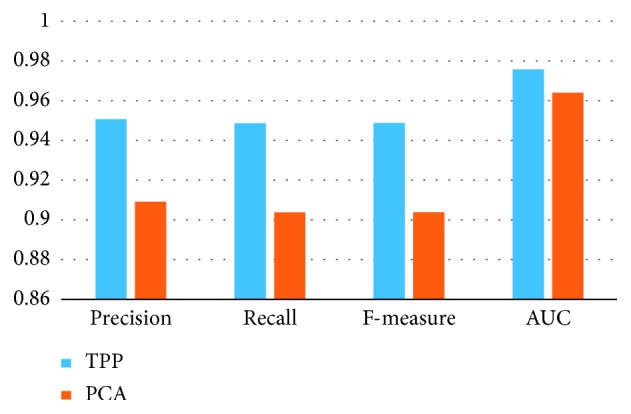
The comparison of PCA and TPP.

In this work, a temporal pyramid pooling method to reduce the dimension of the feature vector is designed. Here, we compare the traditional PCA method and the proposed feature processing method in this experiment. We select 9 relevant features with the MANOVA method and also choose the top 9 principal components in the PCA method to maintain the consistency of feature dimension. The result of this experiment is in Figure 7.

From the result of the experiment, our method can get a precision of 95.1% in recognition of the severity of hypertension while the PCA method can only reach 90.9%. Our method is obviously better than the traditional PCA method. This means that the temporal pyramid pooling method can effectively reserve the discriminant information and benefit for the training of the recognition of the severity of hypertension. Temporal pyramid pooling method can maintain the physical meaning of features and greatly reflect the physiological status of the patients.

## 7. Conclusion

Hypertension is one of the most threatening chronic health problems at present and causes a lot of concurrent diseases. Blood pressure is affected by the autonomous nervous system, and HRV is a good indicator for the autonomous nervous system. Therefore, HRV can be used as an early indicator of hypertension. Early detection is important for better prognosis and prevents the progression of the hypertension. In the present work, an attempt has been made to discriminate the severity of hypertension from heart rate variability during whole-night natural sleep. Based on the long-term ECG data of 139 hypertensive patients, a multiscale segmented HRV analysis was investigated based on the multidimensional 18 features extracted from the time domain, frequency domain, and nonlinear domain. The classification results show that the RF classifier module with the temporal pyramid pooling method and MANOVA has higher precision and recall, which reaches a precision of 95.1%. Hence, the proposed approach is more fine-grained and may offer a better diagnostic way to distinguish the severity of hypertension patients, thereby allowing a more objective assessment and early warning. Further prospective studies with a larger number of participants are necessary to investigate the long-term prognostic significance of the HRV, and the potential HRV changing pattern during the whole night while the patients are in different stages of hypertension.

## Figures and Tables

**Table 1:** Severity of hypertension.

Classes	SBP (mmHg)	DBP (mmHg)	TAGS
Normal	<120	<80	Mild
Normal hypertension	120–139	80–89	Moderate
Level I hypertension	140–159	90–99	

Level II hypertension	160–180	100–109	Severe
Level III hypertension	≥180	≥110	

**Table 2:** Feature dimension of different time scales.

Time scales	Feature dimension
5 min	96
20 min	24
30 min	16
1 hour	8
2 hours	4
4 hours	2
8 hours	1

**Table 3:** Feature dimensions of temporal pyramid data pooling.

Time scale	5 min	20 min	30 min	1 hour	2 hours	4 hours	8 hours	Multiscale
Feature	96	24	16	8	4	2	1	151

**Table 4:** Maximum information entropy and *k* value.

Feature	*K* value	Information entropy
CV	31	1.4631
MAX	148	1.4131
MEAN	133	1.5476
MIN	123	1.5379
PNN50	134	1.4468
RMSSD	57	1.4547
SDNN	10	1.5056
SDSD	21	1.3938
VAR	11	1.5400
HF	150	1.4131
LF	23	1.5608
VP	16	1.4814
LF/HF	2	1.3257
VHF	15	1.4388
VLF	55	1.5520
DFA	7	1.5060
Sample entropy	7	1.3647
Renyi entropy	14	1.5362

**Table 5:** MANOVA of single-scale data pooling.

Features	5 min	20 min	30 min	1 hour
CV	0.211	0.864	0.094	0.923
MAX	2.694	1.293	1.62	1.69
MEAN	1.206	1.368	1.05	1.391
MIN	2.614	0.865	0.986	0.413
PNN50	0.641	0.891	0.124	0.679
RMSSD	0.223	0.779	0.63	1.942
SDNN	0.572	0.671	0.245	0.122
SDSD	0.411	1.307	1.043	1.8
VAR	0.6	0.743	0.303	0.125
HF	0.228	0.432	0.474	1.445
LF	0.965	1.737	0.973	0.702
VP	0.533	0.936	0.338	0.209
LF/HF	2.242	3.562	4.184	2.992
VHF	0.835	1.191	1.319	2.46
VLF	0.884	0.829	0.677	0.57
DFA	0.445	0.046	0.322	0.018
Sample entropy	0.675	0.291	0.444	0.092
Renyi entropy	0.239	0.26	0.117	0.243

**Table 6:** MANOVA of temporal pyramid pooling.

Feature	DOF	F-measure	Feature	DOF	F-measure
CV	2	0.365	HF	2	1.270
MAX	2	1.850	LF	2	1.112
MEAN	2	1.248	VP	2	0.611
MIN	2	0.475	LF/HF	2	3.051
PNN50	2	0.936	VHF	2	1.619
RMSSD	2	1.100	VLF	2	1.760
SDNN	2	0.365	DFA	2	0.160
SDSD	2	1.457	Sample entropy	2	0.920
VAR	2	0.349	Renyi entropy	2	0.558

**Table 7:** Results of different classifiers for three datasets.

Classifier	Naïve Bayes	SVM	BPNN	RF
Mild	0.255	0	0.680	0.919
Moderate	0.588	0.559	0.913	0.959
Severe	0.240	0	0.655	0.952

## Data Availability

The SHAREE (Smart Health for Assessing the Risk of Events via ECG) data used to support the findings of this study have been deposited in the Physionet repository (https://physionet.org/physiobank/database/shareedb/).
